# Supplementary material for: AP-3 and the V-ATPase Modulate CTP Synthase Assembly Through Spatial Association at the Yeast Vacuole
Source: bioRxiv. 2026 Feb 16:2026.02.13.705788. Preprint. [Version 1] doi: 10.64898/2026.02.13.705788 (PMC12934586; doi:10.64898/2026.02.13.705788)
Supplement: Supplement 2 [file media-2.pdf]

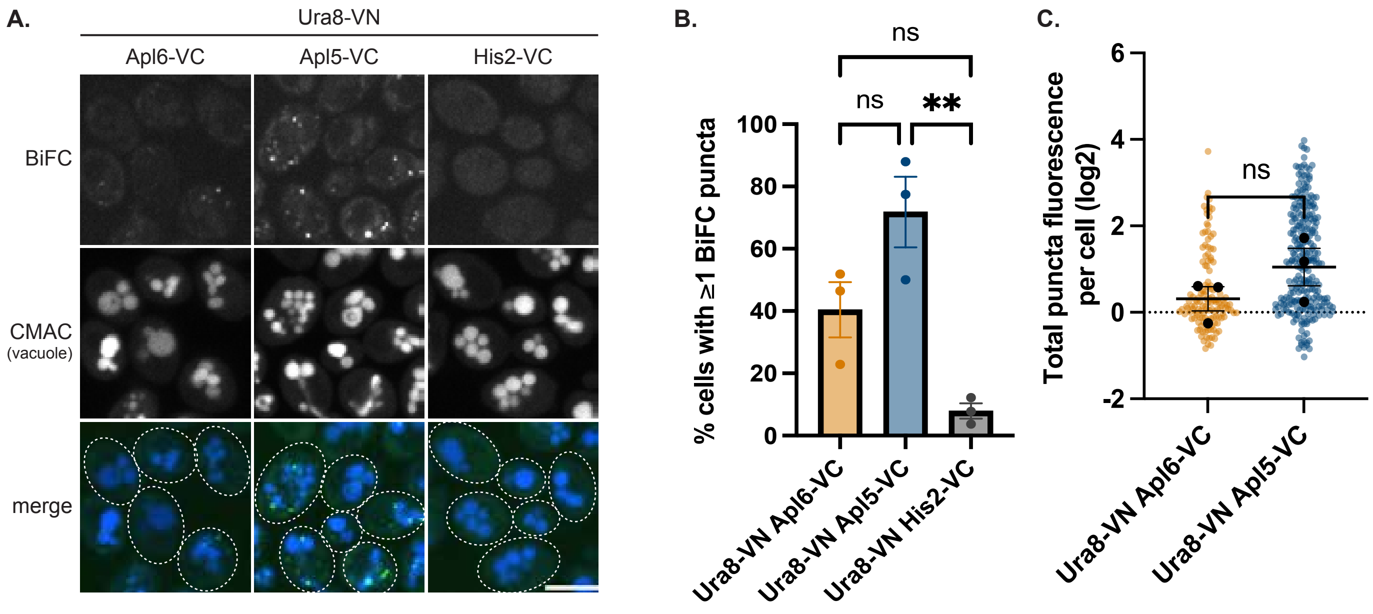

Figure S2. The CTP synthase homolog Ura8 associates with AP-3 by BiFC

(A) Representative confocal images of cells expressing the indicated VN/VC fusions. Scale bar, 5 µm. Quantification of **(B)** BiFC-positive cells **p=0.004; ns, not significant (Ura8-VN His2-VC vs. Ura8-VN Apl6-VC, p=0.077; Ura8-VN His2-VC vs. Ura8-VN Apl6-VC, p=0.086) and **(C)** total BiFC fluorescence. ns, not significant (p=0.229). Graphs and statistics as in Figure 1B,C.
